# Remaining Root Filling Material in Oval Canals After Retreatment Using MicroMega Remover and Reciproc Blue Systems with and Without Passive Ultrasonic Irrigation: A Micro-CT Study

**DOI:** 10.3390/jcm15124822

**Published:** 2026-06-21

**Authors:** Furkan Konus, Faruk Oztekin

**Affiliations:** 1 Department of Endodontics, Faculty of Dentistry, Yozgat Bozok University, Yozgat 66200, Turkey; 2 Department of Endodontics, Faculty of Dentistry, Firat University, Elazığ 23119, Turkey; foztekin@firat.edu.tr

**Keywords:** endodontic retreatment, micro-computed tomography, passive ultrasonic irrigation, remaining filling material

## Abstract

**Background/Objectives**: The aim of this study was to compare the effectiveness of the Reciproc Blue (RB) and MicroMega Remover (MR) systems in removing root canal filling material and to evaluate the effect of passive ultrasonic irrigation (PUI) on remaining filling material (RFM) using micro-computed tomography (micro-CT)-based three-dimensional (3D) analysis. **Methods**: Forty single-rooted mandibular premolar teeth were included in the study. The root canals were prepared up to size F2 using the ProTaper Gold rotary file system and obturated with the lateral compaction technique. After the initial micro-CT scan, the teeth were randomly divided into four groups: Group RB, Group MR, Group RB + PUI, and Group MR + PUI (*n* = 10). Following retreatment, a second micro-CT scan was performed. The percentage of RFM was calculated, and statistical analyses were performed using Kruskal–Wallis and Mann–Whitney U tests with Bonferroni correction. A rank-based factorial analysis was additionally performed (*p* < 0.05). **Results**: RFM was observed in all groups. No significant difference was found between the RB (7.37%) and MR (7.31%) systems (*p* > 0.05). However, the groups treated with PUI (RB + PUI and MR + PUI) showed significantly lower RFM values than the groups without PUI (*p* = 0.001). Factorial analysis revealed no significant effect of file system or file system × PUI interaction, whereas PUI significantly reduced RFM (*p* < 0.001). **Conclusions**: The RB and MR systems demonstrated similar effectiveness in removing root canal filling material. Although complete canal cleanliness could not be achieved, under the in vitro conditions of the present study, PUI significantly reduced the amount of micro-CT-measured RFM.

## 1. Introduction

Although root canal treatment has high success rates, long-term periapical healing is associated with several factors, including the complex anatomy of the root canal system, the adequacy of microorganism elimination, and the effectiveness of the applied disinfection protocols [1,2]. In cases where primary treatment fails, non-surgical endodontic retreatment is a commonly preferred clinical approach for the elimination of persistent or secondary infections [3]. In this context, the success of retreatment largely depends on the effective re-cleaning and disinfection of the root canal system.

One of the main objectives of retreatment is the removal of root canal filling material to the greatest extent possible. Remaining filling materials (RFM) may contribute to the persistence of microorganisms by limiting the penetration of irrigating solutions into the dentinal surface and particularly into anatomically challenging areas such as apical irregularities, isthmuses, and oval-shaped canals [4,5]. Despite the use of different instrumentation techniques, several studies have consistently reported the presence of considerable amounts of RFM on canal walls [5]. These findings suggest that mechanical preparation alone may be insufficient.

To overcome these limitations, recent developments in nickel–titanium (Ni-Ti) systems have focused on improving alloy properties and file design to enhance retreatment performance [6,7]. In this regard, the MicroMega Remover (MR) system (Micro-Mega, Besancon, France) is one of the single-file systems specifically developed for retreatment and features increased flexibility and cyclic fatigue resistance due to C-wire thermal treatment [8]. Its asymmetric cross-sectional design and mechanical properties may facilitate debris removal during retreatment procedures [8]. However, the current literature regarding the MR system is limited, and the clinical significance of its retreatment performance has not yet been fully clarified. Therefore, comparison with a well-established and extensively investigated heat-treated single-file system may provide a more meaningful evaluation of the retreatment performance of this recently introduced system.

Reciproc Blue (RB) system (VDW, Munich, Germany), which has been extensively investigated in the literature, is a heat-treated single-file system with well-documented retreatment performance [9,10,11]. Owing to its enhanced flexibility, increased cyclic fatigue resistance, and widespread use in retreatment research, RB was selected as the reference system for comparison with MR.

Nevertheless, the effectiveness of mechanical instrumentation may remain limited in complex anatomical regions of the root canal system, such as isthmuses and oval-shaped canals [5,12]. Therefore, activation of irrigation has emerged as an important adjunctive factor in improving retreatment success. Passive ultrasonic irrigation (PUI) may contribute to the removal of RFM in inaccessible areas by enhancing the penetration and cleaning ability of irrigating solutions through its strong acoustic streaming effect and the associated potential cavitation [11,13].

Although studies evaluating the combined use of instrumentation systems and irrigation activation methods are available [4,11], the extent to which this effect differs among new-generation single-file systems remains unclear. However, evidence regarding the retreatment performance of the MR system, particularly when combined with PUI and evaluated using micro-computed tomography (micro-CT) analysis, remains limited.

The aim of this study was to compare the effectiveness of the RB and MR systems in removing root canal filling material and to evaluate the effect of PUI on the amount of RFM using micro-CT-based three-dimensional (3D) analysis.

The null hypotheses tested in this study were as follows:

1. There is no significant difference between the file systems used (RB and MR) in terms of the percentage of RFM.
2. There is no significant difference in the percentage of RFM between the groups treated with and without PUI.

## 2. Materials and Methods

### 2.1. Ethical Approval and Sample Size Calculation

This study was approved by the Non-Interventional Research Ethics Committee of Fırat University (decision no: 2022/14-01; date: 1 December 2022) and conducted in accordance with the Declaration of Helsinki. The sample size was determined using G*Power version 3.1 (Heinrich Heine University, Düsseldorf, Germany). An a priori power analysis was performed using the F-test family and the ANOVA: fixed effects, omnibus, one-way test model. The analysis was conducted with an effect size of f = 0.75 [4,14,15], an alpha error probability of 0.05, a statistical power of 90%, and four study groups. The assumed large effect size (f = 0.75) was derived from previous micro-CT retreatment studies reporting substantial differences among retreatment protocols [4,14,15]. The minimum required total sample size was calculated as 36 specimens. Considering balanced group allocation and possible sample loss, a total of 40 mandibular premolar teeth were included in the study, with 10 specimens allocated to each group. The sample-size calculation was based on a one-way omnibus ANOVA model; however, the final statistical analysis additionally included a factorial framework to evaluate the main effects of the retreatment system and PUI, as well as their interaction.

#### Sample Preparation, Inclusion, and Exclusion Criteria

Extracted mandibular premolar teeth were obtained from the Departments of Oral and Maxillofacial Surgery and Periodontology of Fırat University. The teeth had been extracted for periodontal or orthodontic reasons and were anonymized prior to inclusion in the study. The teeth included in the study were evaluated using cone-beam computed tomography (CBCT) images (Romexis Viewer Software, version 6.4.4.7; Planmeca Oy, Helsinki, Finland). Straight, single-rooted, mature teeth with oval-shaped root canals and similar root lengths were included in the study [16]. Canals were classified as oval when the ratio of the buccolingual diameter to the mesiodistal diameter was ≥2 [17]. Teeth with previous endodontic treatment, resorption, carious lesions, cracks, or calcifications were excluded. After selection, all hard and soft tissue remnants were removed using periodontal instruments, and the teeth were stored in physiological saline until use.

### 2.2. Root Canal Preparation and Obturation

To ensure standardization, teeth measuring 20 ± 1 mm in length were selected. Access cavities were prepared using a water-cooled diamond round bur. The working length was determined by subtracting 1 mm from the length at which a size 10 K-file became visible at the apical foramen. No mechanical glide path was established prior to instrumentation; the size 10 K-file was used solely for working length determination. Root canal preparation was performed using the ProTaper Gold (Dentsply Sirona, Ballaigues, Switzerland) system according to the manufacturer’s instructions and recommended motor settings [18]. After enlargement of the coronal third with the SX file, the S1 and S2 files were used with brushing motions up to the working length. Final instrumentation was then completed up to the working length using the F1 and F2 files. Each ProTaper Gold instrument (SX, S1, S2, F1, and F2) was used for the preparation of a single canal only and was discarded thereafter. Between each file change, the canals were irrigated with 2 mL of 5.25% sodium hypochlorite (NaOCl) using a 30-gauge side-vented irrigation needle positioned 1–2 mm short of the working length. Final irrigation consisted of 2 mL of 17% EDTA delivered using the same needle and insertion depth and maintained in the canal for 1 min, followed by 2 mL of distilled water delivered using the same needle and insertion depth. After drying the canals with paper points, the fit of the F2 master gutta-percha cone (Dentsply Maillefer, Ballaigues, Switzerland) was verified at the working length. Obturation was then performed using AH Plus root canal sealer (DeTrey Dentsply, Konstanz, Germany) and the F2 master cone. Following placement of the master cone, lateral compaction was completed using an ISO size 25 spreader and ISO size 20, 2% taper accessory gutta-percha cones. Accessory cones were inserted until the spreader could no longer penetrate beyond the coronal 1–2 mm of the canal. After obturation, the access cavities were restored with glass ionomer cement (Ruby Liner, İnci Dental, Istanbul, Türkiye). All specimens were stored at 37 °C in 100% humidity for 1 month.

### 2.3. Initial Micro-CT Analysis

To determine the root canal filling volume of the specimens, initial micro-CT scans were performed using a micro-CT device (Scanco Medical µCT50, Brüttisellen, Switzerland) at 90 kVp and 155 μA with a 0.1 mm thick copper filter. The voxel size was set at 20 μm, and the integration time was 300 ms. The acquired raw data were reconstructed three-dimensionally using the manufacturer-provided software (Scanco Medical µCT Evaluation Program v6.5, Switzerland). Before segmentation, a Gaussian filter (sigma = 0.8, support = 1) was applied to all reconstructed datasets to reduce image noise while preserving structural boundaries. Quantification of the root canal filling material was performed using threshold-based segmentation. A fixed global threshold was applied to all specimens, with the lower threshold set at 368 and the upper threshold at 1000 (scanner units). These threshold values were selected based on the markedly higher radiopacity of the root canal filling material compared with the surrounding dentin, enabling reliable discrimination of filling material from dental hard tissues and background structures. Quantitative analyses were performed within the same software, and root canal filling volumes were calculated in mm^3^.

### 2.4. Group Allocation

The study was divided into four groups according to the file system used and the use of PUI. All teeth were numbered from 1 to 40 and randomly allocated to the groups using numbers generated by the Google Random Number Generator. Group allocation was performed by an investigator who was not involved in the experimental procedures or outcome assessment.

Randomization was statistically verified, and no significant difference was found among the groups regarding initial root canal filling volumes (mean volume: 10.96 mm^3^, *p* > 0.05), indicating comparable canal filling volumes prior to retreatment procedures.

The groups were established as follows:

- Group RB: Reciproc Blue system;
- Group MR: MicroMega Remover system;
- Group RB + PUI: Reciproc Blue system with PUI;
- Group MR + PUI: MicroMega Remover system with PUI.

### 2.5. Root Canal Retreatment Procedures

#### 2.5.1. Group RB: Reciproc Blue System

Gates-Glidden drills sizes 1 and 2 were used sequentially in the coronal 2–3 mm portion of the root canal. Following coronal enlargement, retreatment was performed directly using the Reciproc Blue RB25 file (VDW, Munich, Germany) without establishing a mechanical glide path. The RB25 file was operated using the X-Smart Plus endomotor (Dentsply Sirona, Ballaigues, Switzerland) in the “RECIPROC ALL” mode. The RB25 file was advanced into the root canal using an in-and-out pecking motion with an amplitude of approximately 3 mm. After every three pecking motions, the instrument was removed from the canal, cleaned with a moist sponge, and the canal was irrigated with 2 mL of 5.25% NaOCl using a 30-gauge side-vented irrigation needle positioned 1–2 mm short of the working length. These procedures were repeated until the working length was reached and no visible filling remnants remained within the canal. Final irrigation was performed sequentially with 5 mL of 5.25% NaOCl, 5 mL of 17% EDTA, and 5 mL of distilled water. The canals were dried with sterile paper points.

#### 2.5.2. Group MR: MicroMega Remover System

After the use of Gates-Glidden drills as described for the RB group, retreatment was performed using the MR file. The MR file was operated using the X-Smart Plus endomotor set at 2.5 Ncm torque and 500 rpm. The instrument was advanced using the same in-and-out pecking motion protocol described for Group RB (approximately 3 mm amplitude). After every three pecking motions, the instrument was removed from the canal, cleaned with a moist sponge, and irrigation was performed using 2 mL of 5.25% NaOCl delivered with a 30-gauge side-vented irrigation needle positioned 1–2 mm short of the working length. These procedures were repeated until the working length was reached and no visible filling remnants remained within the canal. The remaining procedures were identical to those described for Group RB.

#### 2.5.3. Group RB + PUI: Reciproc Blue System with PUI

Following retreatment procedures in Group RB, PUI was subsequently applied. The root canal was filled with 2 mL of 5.25% NaOCl, and a straight, smooth ultrasonic tip (Silver-Tip 20/02; Changzhou Sifary, Changzhou, China) positioned 1–2 mm short of the apex was activated using an ultrasonic activation device (Ultrasonic Activator; Changzhou Sifary, Changzhou, China) in three 20 s cycles (total activation time: 1 min). Fresh 2 mL aliquots of 5.25% NaOCl were used between each cycle, resulting in a total NaOCl volume of 6 mL during ultrasonic activation. The ultrasonic tip was activated using in-and-out movements within the canal. Subsequently, the canals were filled with 17% EDTA and activated for 1 min using the same protocol, with fresh 2 mL aliquots used between cycles. Final irrigation and canal drying were performed as previously described.

#### 2.5.4. Group MR + PUI: MicroMega Remover System with PUI

After completion of root canal filling material removal as described for Group MR, PUI was performed as described for Group RB + PUI.

All specimens were prepared by a single experienced operator. RB25 and MR files were used in a single canal only.

### 2.6. Micro-CT Evaluation of RFM After Retreatment

**Figure 1:**
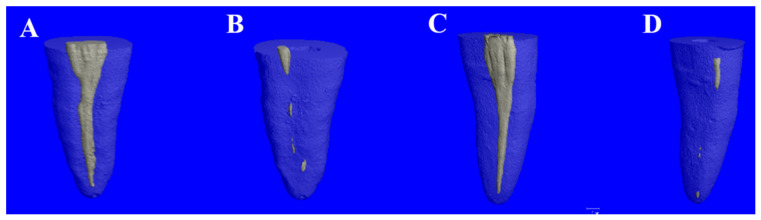
Representative micro-CT images of the MR groups before and after retreatment. (**A**) Initial root canal filling of the MR group; (**B**) remaining filling material after retreatment using the MR system; (**C**) initial root canal filling of the MR + PUI group; and (**D**) remaining filling material in the MR + PUI group after retreatment.

**Figure 2:**
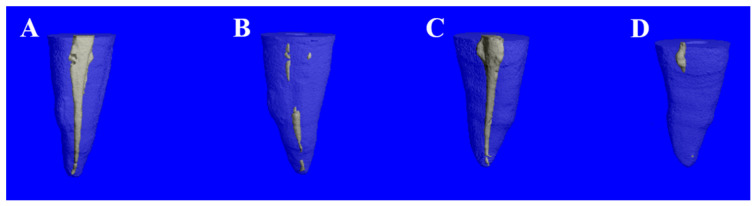
Representative micro-CT images of the RB groups before and after retreatment. (**A**) Initial root canal filling of the RB group; (**B**) remaining filling material after retreatment using the RB system; (**C**) initial root canal filling of the RB + PUI group; and (**D**) remaining filling material in the RB + PUI group after retreatment.

Following the removal of root canal filling material, the specimens were rescanned using the same acquisition parameters applied in the initial micro-CT analysis. The specimens were positioned in accordance with their location and orientation during the initial scan. The acquired data were reconstructed and converted into 3D models using the same software (Scanco Medical µCT Evaluation Program v6.5, Switzerland) as described in the initial analysis. The same Gaussian filtering and threshold-based segmentation parameters (sigma = 0.8, support = 1; threshold range: 368–1000 scanner units) were applied to all post-retreatment scans. Volumetric measurements of RFM were calculated in mm^3^ by a blinded examiner who was unaware of the group allocation during the analysis. The percentage of RFM was determined by dividing the remaining filling volume by the initial filling volume, and the results were expressed as percentages (%). Representative images of the groups before and after retreatment with and without PUI are presented in Figure 1 and Figure 2.

### 2.7. Statistical Analysis

The data were analyzed using IBM SPSS software (IBM Corp., Version 26.0, Armonk, NY, USA). Mean RFM percentage values were calculated for each group. Data distribution was evaluated using the Shapiro–Wilk test. Since the data did not show normal distribution, Kruskal–Wallis and Mann–Whitney U tests were used for inter-group comparisons. Following the Kruskal–Wallis test, pairwise comparisons were performed using Mann–Whitney U tests with Bonferroni correction for multiple comparisons. Because the study had a 2 × 2 factorial design, with file system and PUI use as the two independent factors, the main effects of file system and PUI use and the file system × PUI interaction were additionally evaluated. Since the data were not normally distributed, RFM (%) values were converted into ranks, and a two-way factorial analysis was performed on the ranked data. Statistical significance was set at *p* < 0.05.

## 3. Results

RFM was observed in all groups. A statistically significant difference was found among the groups (*p* = 0.001) (Table 1).

The highest RFM values were observed in the RB (7.37%) and MR (7.31%) groups, whereas the lowest values were observed in the RB + PUI (2.53%) and MR + PUI (2.80%) groups. No significant difference was found between the RB and MR groups; however, both groups showed significantly higher RFM values compared with the PUI-treated groups (*p* < 0.05).

The groups treated with PUI (RB + PUI and MR + PUI) demonstrated significantly lower RFM values compared with the groups without PUI (RB and MR) (*p* = 0.001) (Table 2).

No significant main effect was found for the file system (*p* = 1.000). However, a significant main effect was observed for PUI use (*p* < 0.001). The file system × PUI interaction was not statistically significant (*p* = 0.667) (Table 3).

## 4. Discussion

The presence of RFM in the root canal system may affect the success of retreatment procedures. Therefore, selecting an effective and rapid technique is of great importance for clinicians [19]. In the present study, the effectiveness of the RB and MR systems in removing root canal filling material and the effect of PUI on this process were evaluated. The findings revealed no significant difference between the file systems used, whereas PUI significantly reduced the amount of RFM. Accordingly, the first null hypothesis was accepted, while the second null hypothesis was rejected.

Various methods have been used to evaluate RFM after retreatment, including two-dimensional radiographic imaging [20], CBCT [21], image analysis software [22], scanning electron microscopy [23], and micro-CT. Compared with other methods, micro-CT stands out due to its high resolution, more precise measurement capability, noninvasive nature, and ability to provide 3D evaluation [9,24]. Therefore, micro-CT was preferred in the present study for the 3D and quantitative evaluation of RFM.

Although some researchers have suggested that artificial resin blocks are superior in terms of standardization of the initial root canal filling volume, natural teeth were used in the present study because acrylic blocks may not adequately reflect the complexity of the natural tooth structure and clinical conditions [25]. It has been reported that removal of the crowns of natural teeth is necessary to standardize the working length and access to the root canal filling material [11,26]. However, removal of clinical crowns may not fully represent the actual clinical scenario. Therefore, limiting the length of the root canal filling has been emphasized as a more appropriate approach for working length standardization [25]. Nevertheless, it has also been demonstrated that standardization of the working length and access cavity is possible while preserving the crowns [14]. Accordingly, the crowns were preserved in the present study, preoperative CBCT images were evaluated, and teeth with a length of 20 ± 1 mm were selected. Furthermore, straight single-rooted mandibular premolars with oval canals were selected to improve standardization while preserving a challenging canal anatomy relevant to retreatment procedures. Standardization was achieved by preparing all canals to similar lengths and obturating them with the same apical diameter. The statistically similar initial root canal filling volumes among the groups indicate successful standardization and allow reliable comparison of the obtained results [27].

In the present study, the files were used until the working length was reached, and no additional file was used to evaluate the effectiveness of the single-file systems. File systems differ in metallurgical and design characteristics, including cross-sectional geometry, alloy composition, tip diameter, and taper design. RB has an S-shaped cross-sectional design and is manufactured from a blue heat-treated alloy. In contrast, MR is manufactured using C-wire alloy and has a variable triple-helix cross-sectional geometry. Standardization of these differences is difficult and may influence retreatment performance in oval root canals.

According to the findings of the present study, no statistically significant difference was observed in terms of RFM when the groups without PUI were evaluated (*p* > 0.05). However, Group MR demonstrated slightly lower RFM values than Group RB. This finding is consistent with the studies of Özkan et al. [28], who evaluated ProTaper Universal Retreatment (PTUR), MR, and Hedström files in maxillary incisors, and Dursun et al. [29], who investigated the effectiveness of PTUR, MR, Reciproc, and Hedström files in curved root canals. However, direct comparison should be interpreted with caution because only a single-file system was used and no supplementary apical enlargement was performed.

Dedeus et al. [27] reported that the use of additional files and increased apical preparation size significantly enhanced root canal filling removal, whereas Madarati et al. [11] stated that file size and taper did not affect the effectiveness of root canal filling removal. In the present study, RB (25/0.08) and MR (30/0.07) files, which differed in tip size and taper, demonstrated similar effectiveness and showed very close RFM values (7.37% and 7.31%, respectively). From a theoretical perspective, the larger tip size of the MR file (30/0.07) could be expected to facilitate greater contact with canal walls and enhance the removal of filling remnants, whereas the greater taper of the RB file (25/0.08) may increase its cutting efficiency in the coronal and middle thirds of the canal. Therefore, both instruments possess design characteristics that may influence retreatment performance in different ways. The similar RFM values observed in the present study suggest that these differences may have balanced each other under the experimental conditions employed. Similarly, Versiani et al. [30] demonstrated that although progressive canal enlargement reduced the amount of unprepared canal walls, complete preparation of anatomically complex canal systems could not be achieved, while dentine removal and the risk of procedural complications increased with larger preparations. Although that study evaluated canal preparation rather than retreatment procedures, its findings may help explain why complete removal of root canal filling material remains challenging despite enlargement procedures. In addition, previous studies have demonstrated that thermal treatment may substantially influence the flexibility and mechanical behavior of Ni-Ti instruments [31,32]. However, instrument performance is multifactorial and depends not only on alloy properties but also on cross-sectional design, taper, core diameter, cutting efficiency, and kinematic motion [32]. Therefore, enhanced flexibility alone does not necessarily result in superior filling material removal. This may partly explain why the RB and MR systems demonstrated similar RFM values despite their different metallurgical characteristics and design features. Although the RFM values observed in the present study were consistent with most previous studies by remaining below 10% [9,11], they were still above the 0.5% RFM value proposed as the cutoff point for “effective canal cleaning” [33]. Spinelli et al. [34] reported that RFM in oval canals was predominantly located in the middle third and buccolingual extensions of the canal despite retreatment procedures, emphasizing the difficulty of achieving complete cleanliness in oval canal anatomies. These findings suggest that complete removal of root canal filling material remains challenging despite contemporary instrumentation systems. Therefore, irrigation activation procedures may provide an important adjunctive contribution to the removal of RFM during retreatment [35].

Nevertheless, conflicting results regarding the effectiveness of PUI in the removal of root canal filling materials have been reported in the literature [11,14,36]. These differences may be related to variations in root canal anatomy, filling materials, and study protocols. In the present study, the amount of RFM was significantly lower in the groups treated with PUI. This finding may be associated with the preparation method of the tooth specimens and the anatomy of the root canals. Although easier access to the root canal filling material in decoronated teeth may improve retreatment outcomes [37,38], removal of the coronal portion may negatively affect the activation and flow of irrigating solutions due to the reduced irrigant reservoir volume [39]. Therefore, preservation of the coronal portion of the teeth in the present study may have contributed to the effectiveness of PUI by providing a reservoir area for the irrigating solution. However, RFM was detected in all groups, indicating that complete retreatment cleanliness could not be achieved even when PUI was used. The clinical significance of this reduction should be interpreted with caution, as the present study evaluated RFM using a micro-CT model and did not assess clinical outcomes such as microbial reduction, periapical healing, or long-term treatment success. Therefore, while the reduction in RFM observed in the present study may be considered advantageous from a retreatment perspective, its direct impact on clinical outcomes remains to be confirmed by future clinical studies.

The absence of solvent use in the present study may be considered a limitation. However, the role of solvents in enhancing root canal filling removal remains controversial [40,41,42]. Previous studies have shown that solvents do not significantly improve canal cleanliness and may lead to the accumulation of gutta-percha and sealer remnants within canal irregularities and dentinal tubules [40,43]. Therefore, solvent use was intentionally avoided in the present study.

Procedural errors are among the undesirable events during retreatment, and no file fracture occurred in the present study. Previous studies have reported that different heat-treatment procedures increase the cyclic fatigue resistance and flexibility of Ni-Ti files, which may provide advantages in canals with complex anatomy [29,31,44]. This may explain the absence of file fracture during retreatment procedures. However, since only straight root canals were included, caution is required when extrapolating these findings to curved canals. Further studies evaluating their performance in curved canals are warranted.

This study has several limitations. The study was conducted under in vitro conditions using single-rooted teeth and may not fully reflect clinical variations. In addition, only straight mandibular premolars with single oval canals were included, and all canals were obturated using the lateral compaction technique. These conditions may have facilitated retreatment procedures compared with more challenging clinical situations involving curved canals, multi-rooted teeth, complex canal anatomies, or different obturation techniques. Therefore, caution should be exercised when extrapolating the present findings directly to routine clinical practice. In addition, the distribution of RFM within the coronal, middle, and apical thirds was not evaluated [34]. Retreatment procedures were completed when the working length was reached and no visible filling remnants remained. However, RFM may persist within inaccessible extensions of oval canals despite the canal appearing clinically clean. Furthermore, no standardized supplementary apical enlargement was performed after filling removal, which may have influenced the amount of RFM by limiting further mechanical cleaning and irrigant penetration. In addition, the PUI groups received supplementary irrigation and activation procedures after mechanical retreatment. Therefore, it cannot be determined whether the observed reduction in RFM was attributable solely to ultrasonic activation or partly to the increased irrigant volume, contact time, and chemical action. Future studies should include control groups with equivalent irrigation protocols but without ultrasonic activation to better isolate the effect of PUI. Retreatment working time was not recorded; therefore, the efficiency of the tested systems and the additional time required for PUI could not be evaluated. Nevertheless, the findings provide important information regarding the comparative performance of the tested systems. Future studies including larger sample sizes, clinical designs, and combined evaluation of irrigation activation methods are recommended.

## 5. Conclusions

Remaining root canal filling material was observed in all groups after retreatment, regardless of the file system and irrigation protocol used. The RB and MR systems demonstrated similar effectiveness in removing root canal filling material. Although complete canal cleanliness could not be achieved, under the in vitro conditions of the present study, PUI significantly reduced the amount of micro-CT-measured RFM.

## Figures and Tables

**Table 1:** RFM (%) values of the groups obtained by micro-CT analysis.

Groups	*n*	Mean RFM (%)	SD	Mean Rank	Median (IQR)	*p*
RB	10	7.37	2.07	29.10 ^A^	6.99 (2.72)	0.001
MR	10	7.31	2.48	27.90 ^A^	6.36 (2.15)	
RB + PUI	10	2.53	2.17	11.90 ^B^	1.89 (3.17)	
MR + PUI	10	2.80	3.49	13.10 ^B^	1.39 (3.77)	

Different uppercase letters indicate statistically significant differences between groups (*p* < 0.05). SD: standard deviation.

**Table 2:** RFM (%) values in groups with and without PUI.

Groups	*n*	Mean RFM (%)	SD	Mean Rank	Median (IQR)	*p*
MR and RB	20	7.34	2.23	28.50 ^A^	6.67 (2.24)	0.001
RB + PUI and MR + PUI	20	2.67	2.83	12.50 ^B^	1.39 (3.02)	

Different uppercase letters indicate statistically significant differences between groups (*p* < 0.05). SD: standard deviation.

**Table 3:** Rank-based 2 × 2 factorial analysis of RFM (%) values.

Groups	df	F	*p*
File system	1	0.00	1.00
PUI	1	33.45	0.001 *
File system × PUI	1	0.19	0.667

* The analysis was performed on ranked RFM (%) values. *p* < 0.05. df: degree of freedom.

## Data Availability

The original contributions presented in this study are included in the article. Further inquiries can be directed to the corresponding author.

## Abbreviations

The following abbreviations are used in this manuscript:

RB	Reciproc Blue
MR	MicroMega Remover
PUI	Passive ultrasonic irrigation
RFM	Remaining filling material
Micro-CT	Micro-computed tomography
3D	Three-dimensional
CBCT	Cone-beam computed tomography
NaOCl	Sodium hypochlorite
Ni-Ti	Nickel–Titanium

## References

[1] Siqueira J.F., Rôças I.N. (2008). Clinical Implications and Microbiology of Bacterial Persistence after Treatment Procedures. J. Endod..

[2] Ng Y.L., Mann V., Gulabivala K. (2011). A Prospective Study of the Factors Affecting Outcomes of Nonsurgical Root Canal Treatment: Part 1: Periapical Health. Int. Endod. J..

[3] Torabinejad M., Corr R., Handysides R., Shabahang S. (2009). Outcomes of Nonsurgical Retreatment and Endodontic Surgery: A Systematic Review. J. Endod..

[4] Bueno C.E.S., Rios M.A., Coelho M.S., Villela A.M., de Martin A.S., Kato A.S., Alves V.O., Cunha R.S. (2017). Influence of Passive Ultrasonic Irrigation on the Removal of Root Canal Filling Material in Straight Root Canals. Eur. Endod. J..

[5] Alves F.R.F., Rôças I.N., Provenzano J.C., Siqueira J.F. (2022). Removal of the Previous Root Canal Filling Material for Retreatment: Implications and Techniques. Appl. Sci..

[6] Liang Y., Yue L. (2022). Evolution and Development: Engine-Driven Endodontic Rotary Nickel-Titanium Instruments. Int. J. Oral Sci..

[7] Haapasalo M., Shen Y. (2013). Evolution of Nickel–Titanium Instruments: From Past to Future. Endod. Top..

[8] Nehme W., Mallet J., Soulages B., Michel M., Diemer F. (2020). Remover: The Ultimate Solution for Removing Gutta-Percha. Roots Int. Mag. Endod..

[9] Faus-Matoses V., Pasarín-Linares C., Faus-Matoses I., Foschi F., Sauro S., Faus-Llácer V.J. (2020). Comparison of Obturation Removal Efficiency from Straight Root Canals with ProTaper Gold or Reciproc Blue: A Micro-Computed Tomography Study. J. Clin. Med..

[10] Kırıcı D., Demirbuga S., Karataş E. (2020). Micro-Computed Tomographic Assessment of the Residual Filling Volume, Apical Transportation, and Crack Formation after Retreatment with Reciproc and Reciproc Blue Systems in Curved Root Canals. J. Endod..

[11] Madarati A.A., Sammani A.M.N., Alnazzawi A.A., Alrahlah A. (2023). Efficiency of the New Reciprocating and Rotary Systems with or without Ultrasonics in Removing Root-Canals Filling with Calcium Silicate-Based Sealer (MTA). BMC Oral Health.

[12] Sinsareekul C., Hiran-Us S. (2022). Comparison of the Efficacy of Three Different Supplementary Cleaning Protocols in Root-Filled Teeth with a Bioceramic Sealer after Retreatment—A Micro-Computed Tomographic Study. Clin. Oral Investig..

[13] van der Sluis L.W., Versluis M., Wu M.K., Wesselink P.R. (2007). Passive Ultrasonic Irrigation of the Root Canal: A Review of the Literature. Int. Endod. J..

[14] Bernardes R.A., Duarte M.A.H., Vivan R.R., Alcalde M.P., Vasconcelos B.C., Bramante C.M. (2016). Comparison of three retreatment techniques with ultrasonic activation in flattened canals using micro-computed tomography and scanning electron microscopy. Int. Endod. J..

[15] Gomes N.N., de Carvalho G.M., Júnior E.C.S., Garcia L., Marques A.A.F., de Carvalho F.M.A. (2017). Filling Material Removal with Reciprocating and Rotary Systems Associated with Passive Ultrasonic Irrigation. Eur. Endod. J..

[16] Schneider S.W. (1971). A Comparison of Canal Preparations in Straight and Curved Root Canals. Oral Surg. Oral Med. Oral Pathol..

[17] Martins M.P., Duarte M.A.H., Cavenago B.C., Kato A.S., da Silveira Bueno C.E. (2017). Effectiveness of the ProTaper Next and Reciproc Systems in Removing Root Canal Filling Material with Sonic or Ultrasonic Irrigation: A Micro-Computed Tomographic Study. J. Endod..

[18] Sirona D. (2017). ProTaper Gold Rotary Files: Directions for Use.

[19] Yılmaz F., Koç C., Kamburoğlu K., Ocak M., Geneci F., Uzuner M.B., Çelik H.H. (2018). Evaluation of 3 Different Retreatment Techniques in Maxillary Molar Teeth by Using Micro-Computed Tomography. J. Endod..

[20] Serefoglu B., Kandemir Demirci G., Miçooğulları Kurt S., Kaşıkçı Bilgi İ., Çalışkan M.K. (2021). Impact of Root Canal Curvature and Instrument Type on the Amount of Extruded Debris during Retreatment. Restor. Dent. Endod..

[21] Adel M., Tofangchiha M., Rashvand E., Moutabha I., Roohi N., Reda R., Testarelli L. (2022). Comparison of the Efficacy of NeoNiTi, ProTaper, and Reciproc Files in the Retreatment of Curved Root Canals: A CBCT Assessment. Acta Stomatol. Croat.

[22] Zuolo A.S., Mello J.E., Cunha R.S., Zuolo M.L., Bueno C.E. (2013). Efficacy of Reciprocating and Rotary Techniques for Removing Filling Material during Root Canal Retreatment. Int. Endod. J..

[23] Keleş A., Şimşek N., Alçin H., Ahmetoglu F., Yologlu S. (2014). Retreatment of Flat-Oval Root Canals with a Self-Adjusting File: An SEM Study. Dent. Mater. J..

[24] Fischer B.V., Goulart T.S., Vitali F.C., de Souza D.L., Teixeira C.D.S., Garcia L. (2024). Supplementary Methods for Filling Material Removal: A Systematic Review and Meta-Analysis of Micro-CT Imaging Studies. J. Dent..

[25] Ajina M.A., Shah P.K., Chong B.S. (2022). Critical Analysis of Research Methods and Experimental Models to Study Removal of Root Filling Materials. Int. Endod. J..

[26] Rios M.A., Villela A.M., Cunha R.S., Velasco R.C., de Martin A.S., Kato A.S., Bueno C.E.S. (2014). Efficacy of 2 Reciprocating Systems Compared with a Rotary Retreatment System for Gutta-Percha Removal. J. Endod..

[27] De-Deus G., Belladonna F.G., Zuolo A.S., Simões-Carvalho M., Santos C.B., Oliveira D.S., Cavalcante D.M., Silva E.J.N.L. (2019). Effectiveness of Reciproc Blue in Removing Canal Filling Material and Regaining Apical Patency. Int. Endod. J..

[28] Büyüközer Özkan H., Çimen T., Kaya Apaydın S., Er K. (2025). Efficacy of Two Different Retreatment Techniques in Removing Gutta-Percha from Root Canals: A CBCT Study. Turk. Endod. J..

[29] Dursun P.H., Sevimay F.S., Buyuksungur A., Celikten B. (2026). Evaluation of the Effectiveness of MicroMega Remover, ProTaper Universal Retreatment, Reciproc, and Hedstrom Files in the Retreatment of Curved Root Canals Obturated with Different Techniques: A Micro-Computed Tomography Study. Medicina.

[30] Versiani M.A., Carvalho K.K., Martins J.N., Custódio A.L., Castro M.A., Akaki E., Silva-Sousa Y.T., Sousa-Neto M.D. (2022). Effects of root canal enlargement on unprepared areas and coronal dentine thickness of three-rooted maxillary first premolars with different root configurations: A stepwise micro-CT study. Int. Endod. J..

[31] Topçuoğlu H.S., Topçuoğlu G. (2017). Cyclic Fatigue Resistance of Reciproc Blue and Reciproc Files in an S-Shaped Canal. J. Endod..

[32] Silva E.J.N.L., Peña-Bengoa F., Ajuz N.C., Vieira V.T., Martins J.N., Marques D., Pinto R., Rito Pereira M., Braz-Fernandes F.M., Versiani M.A. (2024). Multimethod analysis of large- and low-tapered single file reciprocating instruments: Design, metallurgy, mechanical performance, and irrigation flow. Int. Endod. J..

[33] Solomonov M., Paqué F., Kaya S., Adigüzel O., Kfir A., Yiğit-Özer S. (2012). Self-Adjusting Files in Retreatment: A High-Resolution Micro-Computed Tomography Study. J. Endod..

[34] Spinelli A., Zamparini F., Buonavoglia A., Pisi P., Gandolfi M.G., Prati C. (2022). Reciprocating system for secondary root canal treatment of oval canals: CBCT, X-rays for remnant detection and their identification with ESEM and EDX. Appl. Sci..

[35] Güngördü Z.S., Tufenkci P., Sarı M. (2025). The Effectiveness of EndoActivator, EDDY, Passive Ultrasonic Irrigation, and XP-Endo Finisher R in Removing Filling Materials from Retreated Root Canals. BMC Oral Health.

[36] da Rosa R.A., Santini M.F., Cavenago B.C., Pereira J.R., Duarte M.A.H., Só M.V. (2015). Micro-CT Evaluation of Root Filling Removal after Three Stages of Retreatment Procedure. Braz. Dent. J..

[37] Rödig T., Hausdörfer T., Konietschke F., Dullin C., Hahn W., Hülsmann M. (2012). Efficacy of D-RaCe and ProTaper Universal Retreatment NiTi Instruments and Hand Files in Removing Gutta-Percha from Curved Root Canals—A Micro-Computed Tomography Study. Int. Endod. J..

[38] Borges M.M.B., Duque J.A., Zancan R.F., Vivan R.R., Bernardes R.A., Duarte M.A.H. (2019). Efficacy of Reciprocating Systems for Removing Root Filling Material plus Complementary Cleaning Methods in Flattened Canals: Microtomography and Scanning Electron Microscopy Study. Microsc. Res. Tech..

[39] Gündüz H., Özlek E. (2023). The Effects of Laser and Ultrasonic Irrigation Activation Methods on Smear and Debris Removal in Traditional and Conservative Endodontic Access Cavities. Lasers Med. Sci..

[40] Sağlam B.C., Koçak M.M., Türker S.A., Koçak S. (2014). Efficacy of Different Solvents in Removing Gutta-Percha from Curved Root Canals: A Micro-Computed Tomography Study. Aust. Endod. J..

[41] Sae-Lim V., Rajamanickam I., Lim B.K., Lee H.L. (2000). Effectiveness of ProFile. 04 Taper Rotary Instruments in Endodontic Retreatment. J. Endod..

[42] Colombo A.P.M., Fontana C.E., Godoy A., de Martin A.S., Kato A.S., Rocha D.G.-P., Pelegrine R.A., Bueno C.E.S. (2016). Effectiveness of the WaveOne and ProTaper D Systems for Removing Gutta-Percha with or without a Solvent. Acta Odontol. Latinoam..

[43] Ma J., Al-Ashaw A.J., Shen Y., Gao Y., Yang Y., Zhang C., Haapasalo M. (2012). Efficacy of ProTaper Universal Rotary Retreatment System for Gutta-Percha Removal from Oval Root Canals: A Micro-Computed Tomography Study. J. Endod..

[44] Özkan H.B., Sürme K., Akman H., Er K. (2023). Comparison of Cyclic Fatigue Resistance between Heat-Treated and Conventional Retreatment Files. Rev. Port. Estomatol. Med. Dent. Cir. Maxilofac..

